# Probiotics alter biofilm formation and the transcription of *Porphyromonas gingivalis* virulence-associated genes

**DOI:** 10.1080/20002297.2020.1805553

**Published:** 2020-08-20

**Authors:** Karin Hitomi Ishikawa, Daniela Mita, Dione Kawamoto, Jacques Robert Nicoli, Emmanuel Albuquerque-Souza, Maria Regina Lorenzetti Simionato, Marcia Pinto Alves Mayer

**Affiliations:** a Department of Microbiology, Institute of Biomedical Sciences, University of São Paulo, São Paulo, Brazil; b Department of Microbiology, Biological Science Institute, Federal University of Minas Gerais, Belo Horizonte, Brazil; c Division of Periodontics, Department of Stomatology, School of Dentistry, University of São Paulo, São Paulo, Brazil

**Keywords:** Probiotics, periodontitis, gingipain, fimbriae, gene expression, biofilm

## Abstract

**Background and Objective:**

The potential of probiotics on the prevention and control of periodontitis and other chronic inflammatory conditions has been suggested. *Lactobacillus* and *Bifidobacterium* species influence *P. gingivalis* interaction with gingival epithelial cells (GECs) but may not act in a unique way. In order to select the most appropriate probiotic against *P. gingivalis*, we aimed to evaluate the effect of several strains on *Porphyromonas gingivalis* biofilm formation and transcription virulence-associated factors (PgVAFs).

**Methods:**

Cell-free pH neutralized supernatants (CFS) and living *Lactobacillus* spp. and *Bifidobacterium* spp. were tested against *P. gingivalis* ATCC 33277 and W83, in mono- and multi-species (with *Streptococcus oralis* and *S. gordonii*) biofilms. Relative transcription of *P. gingivalis* genes (*fimA, mfa1, kgp, rgp, ftsH* and *luxS*) was determined in biofilms and under GECs co-infection.

**Results:**

Probiotics CFS reduced *P. gingivalis* ATCC 33277 levels in mono-species biofilms and living probiotics reduced *P. gingivalis* abundance in multi-species biofilms. *L. acidophilus* LA5 down-regulated transcription of most PgVAFs in biofilms and GECs.

**Conclusions:**

Probiotics affect *P. gingivalis* biofilm formation by down-regulating overall PgVAFs with the most pronounced effect observed for *L. acidophilus* LA5.

## Introduction

Periodontitis is a chronic inflammatory disease in response to a polymicrobial dysbiotic biofilm that affects supporting periodontal tissues surrounding the teeth and leads to alveolar bone resorption [1]. *Porphyromonas gingivalis* is considered a keystone pathogen in periodontitis due to its properties to trigger an exaggerated pro-inflammatory response that dictates the periodontal destruction, and to evade host defense mechanisms [2]. At the same time, *P. gingivalis* drives a shift in the microbial composition towards a dysbiotic-related state [3].

The current conventional periodontal treatment is based on mechanical debridement of the biofilm plus the use of antimicrobial drugs, aiming to alter the microbial community composition of oral sites, thus leading to control the inflammatory response [4]. However, recent evidences [5] highlight the limitations of this approach and shed light on the search for new therapies. Thus, a proposal to guide periodontal pocket recolonization by the use of probiotics has been raised as a promising strategy [6], as suggested by experimental and clinical studies [5–8]. Meanwhile, despite the evidences showing the benefits of probiotics on the control of the dysbiotic resident microbiota [7,8], little is known on their effects on periodontopathogens.

We have previously shown that probiotic strains of the genera *Lactobacillus* and *Bifidobacterium* promote a reduction in *P. gingivalis* adhesion to and invasion of gingival epithelial cells, and alter the cell response to the pathogen [9]. These beneficial microorganisms may also directly exert an effect on certain pathogens by attenuating their virulence components [10], such as by impairing toxins production [11]. Regarding oral pathogens, lactobacilli may attenuate the virulence of *Candida albicans* by inhibiting yeast-hypha differentiation [12], and of *Aggregatibacter actinomycetemcomitans* by down-regulating the expression of exotoxins [13]. Nonetheless, little is known about the effects of interactions between probiotics and the keystone pathogen *P. gingivalis*.

A successful colonization of the oral cavity by *P. gingivalis* depends on its ability to interact with early colonizers in the dental biofilm, e.g. *Streptococcus oralis* and *S. gordonii* [14,15], to adhere to and invade the epithelial barrier [15–17], and to evade host immunity [18]. Despite intra-species diversity, *P. gingivalis* pathogenic potential is associated with factors such as the expression of capsule, the main (FIMA) and minor (MFA1) fimbriae [19], lysine-(KGP) and arginine-(RGP) gingipains [20], quorum sensing components (LUXS) [21], and metallopeptidases (FTSH) [22].

Thus, we aimed to investigate whether *Lactobacillus* and *Bifidobacterium* probiotics would interfere in *P. gingivalis* biofilm formation, by using mono- and multi-species *in vitro* models. Since microbial interactions exert a profound effect on gene expression in a strain-specific fashion [23], we also evaluated the effects of probiotics on the transcription profiles of two *P. gingivalis* strains in biofilms and after gingival epithelial cells co-infection.

## Material and methods

### Strains and culture conditions

Six probiotic strains were tested [*Lactobacillus acidophilus* LA-5™ (CHR Hansen Holding A/S, Hørsholm, Denmark), *L. rhamnosus* HN001 Howaru™ (Danisco, Madison, WI, USA), *L. reuteri* DSM 17938 (BioGaia AB, Lund, Sweden), *Bifidobacterium breve* 110^1A^, *B. pseudolongum* 119^1A^ and *B. bifidum* 162^2A^ (isolated from feces of healthy children, obtained at the Federal University of Minas Gerais) [24]. *P. gingivalis* (W83 and ATCC 33277) were used as periodontopathogens. *S. oralis* ATCC10557 and *S. gordonii* DL1 [25] were used in multi-species biofilm assays.

Bacteria were cultivated from frozen stocks at −80°C. Lactobacilli were grown on Lactobacilli MRS agar (Difco Laboratories, Detroit, MI, USA) and streptococci were cultivated on Tryptic Soy agar [TSA] (Difco Laboratories), both under microaerophilic conditions at 10% CO_2_, 37°C. Bifidobacteria were grown on BSM agar (Bifidus Selective Medium, Sigma-Aldrich, St. Louis, MO, USA) at 37°C, under anaerobic conditions (90% N_2_, 5% CO_2_ and 5% H_2_) in an anaerobe chamber (Plas-Labs Model 855, Lansing, MI, USA). *P. gingivalis* were also grown under anaerobic conditions (90% N_2_, 5% CO_2_ and 5% H2) at 37°C on blood agar plates [TSA (Difco Laboratories) enriched with 5% defibrinated sheep blood, 0.5 mg/mL hemin (Sigma-Aldrich) and 1 mg/mL menadione (Sigma-Aldrich)].

### Cell-free pH neutralized supernatants of probiotic cultures

After growth overnight in Brain-Heart Infusion broth (Neogen, Lansing, MI, USA) supplemented with 5 mg/mL hemin and 10 mg/mL menadione (BHIHM), probiotics cultures were centrifuged at 8,000x*g* for 10 min, and cells resuspended in BHIHM to an OD_600nm_ equivalent to 10^8^ colony forming units (CFU)/mL, diluted 1:10 and incubated under agitation (80 rpm) for 24 h under anaerobiosis. Then, bacteria were removed by centrifugation and the pH of supernatants adjusted to 7.0 with 0.1 M NaOH. Cell-free pH neutralized supernatants (CFS) were sterilized by filtration (0.22 μm pore, Life Sciences, Ann Arbor, MI, USA) and used in biofilm assays.

### Effect of CFS and living cells on biofilm formation

Biofilms were formed in polystyrene 96 wells/plates (Corning Incorporated Costar®, Kennebunk, ME, USA). For mono-species biofilm, aliquots of overnight grown culture in BHIHM of each *P. gingivalis* strain (1 × 10^7^ CFU/well) were inoculated to a total volume of 100 μL. For multi-species biofilm, *P. gingivalis, S. oralis* and *S. gordonii* were inoculated at 1 × 10^7^ CFU/well of each species. In order to evaluate the effect of probiotics CFS on biofilm formation, a final dilution of 1:2.5 of CFS of each probiotic strain was added to BHIHM. In order to evaluate the effect of living probiotics cells on biofilm formation, each probiotic strain was inoculated at 1 × 10^7^ CFU/well. Negative control consisted of non-inoculated medium, and positive controls consisted of mono- or multi-species biofilm without CFS or without living probiotics.

Plates were incubated for 24 h under agitation (80 rpm) on a shaking platform (Sunflower Mini-Shaker, Biosan, Riga, Latvia) in anaerobiosis (90% N_2_, 5% CO_2_ and 5% H2) at 37°C using an anaerobe chamber (Plas-Labs). Biofilm biomass was estimated as previously reported [26]. Briefly, non-adherent bacteria were removed by washing with 1 X Phosphate Buffered Saline (PBS, pH 7.4), and biofilms were stained with 0.4% safranin for 15 min. Excess of dye was removed by washing with distilled water, followed by dye extraction with 95% ethanol for 15 min, and absorbance measured at 490 nm, which represented the biomass amount. All assays were performed in triplicate and independently repeated at least twice.

### *Quantification of* P. gingivalis, S. oralis, S. gordonii, Lactobacillus *spp. and* Bifidobacterium *spp. by qPCR*

DNA in biofilms was extracted using Master Pure^TM^ DNA Purification Kit (Epicentre, Madison, WI, USA), and treated with RNAse. Amplification of *P. gingivalis, S. oralis, S. gordonii*, lactobacilli, and bifidobacteria *16SrRNA* was performed with 0.5 µL of each primer (25 pmol) (Table 1), 10 µL of Power SYBR Green PCR Master Mix (Applied Biosystems, Foster City, CA, USA), 1 µL DNA (10 ng/µL), in a final volume of 20 µL and qPCR was performed by StepOne Plus (Applied Biosystems). Temperature profiles consisted of denaturation (95°C for 10 min), amplification and quantification (40 cycles of 95°C for 15 s and 60°C for 1 min) and melting curve (95°C for 15 s, 60°C for 1 min and heating rate of 0.3°C-95°C for 15 s). For absolute quantification and estimation of PCR efficiency, a standard curve was generated using *16SrRNA* amplicons. Reactions with standards and tested DNA were performed in duplicate. Ct values were correlated with *16SrRNA* copy numbers, and data expressed as the number of each species/well, considering four copies of *16SrRNA* gene per chromosome [27].

### *Interaction of* P. gingivalis *and probiotics with gingival epithelial cells*

The assays were performed according to our previous work [9]. Briefly, immortalized human gingival epithelial cells (OBA-9/GECs) were cultured in Keratinocyte-Serum Free Medium (KSFM) (GIBCO™, Life Technologies, Carlsbad, CA, USA) supplied with human recombinant epidermal growth factor. GECs were seeded in 24-well culture plates at a cell density of 2.0 × 10^5^ cells per well, in KSFM without antibiotic. After 24 h, GECs were challenged with *P. gingivalis* strains and/or probiotics at a multiplicity of infection (MOI) of 1:1,000. After 2 h incubation, unattached bacteria were removed by washing, and GECs were lysed followed by RNA extraction.

### *Effect of probiotics on gene expression of* Porphyromonas gingivalis *VAFs*

Total RNA in adherent biofilms and co-infected epithelial cells were extracted using RNeasy KIT (QIAGEN, Valencia, CA, USA). The quality and concentration of the extracted RNA were determined by measurement of absorbance at 260 and 280 nm in a NanoDrop™ One Spectrophotometer (Thermo Scientific, Waltham, MA, USA). After RNase-free DNase I treatment (Invitrogen Life Technologies), cDNA was synthesized, using 5X VILO^TM^ and 10X SuperScript^TM^ Enzyme (Invitrogen Life Technologies, Waltham, MA, USA) in a GeneAmp PCR System 2400 thermocycler (Applied Biosystems®) set at 25°C for 10 min, 42°C for 60 min and 85°C for 5 min. Control reactions using no enzyme were performed. Gene expression was evaluated with the primers listed in Table 1. Reactions consisted of 0.5 µL of each primer (25 pmol), 10 µL of Power SYBR Green PCR Master Mix (Applied Biosystems®), 1 µL of cDNA at a concentration of 40 ng/µL, completed to a final volume of 20 µL. The RT-qPCR reaction consisted of an activation step of denaturation program (95°C for 10 min), and 40 cycles (95°C for 15 s, 60°C for 1 min with a single fluorescence measurement), melting curve program (95°C for 15 s, 60°C for 1 min, with a heating rate of 0.3°C-95°C for 15 s and a continuous fluorescence measurement) in a StepOne Plus thermocycler (Applied Biosystems®). The efficiency for both the internal control (*16SrRNA*) and gene of interest (GOI) was determined using *P. gingivalis* ATCC 33277 and W83 cDNA templates dilution series as standard curve. Comparative quantification was determined by calculating the C_t_ difference between the target gene in the test and calibrator samples (assays without probiotics CFS or living cells), normalized to the reference gene C_t_ values and adjusted for variations in amplification efficiency [28].

**Table 1.** Oligonucleotides primers for real-time PCR assays used for bacteria quantification in biofilms and for transcription analysis of *P. gingivalis* virulence-associated genes.

Target Gene	Primer sequence (5ʹ-3ʹ)	Reference
*16SrRNA*		
*P. gingivalis*	TGTAGATGACTGATGGTGAAAACC ACGTCATCCCCACCTTCCTC	[60]
*Lactobacillus*	AGCAGTAGGGAATCTTCCA CACCGCTACACATGGAG	[61]
*Bifidobacterium*	TCGCGTC(C/T)GGTGTGAAAG CCACATCCAGC(A/G)TCCAC	[61]
*S. oralis*	CCGCATAAGAGTAGATGTTG TATGTATCGTTGCCTTGGT	[62]
*S. gordonii*	GCTTGCTACACCATAGACT CCGTTACCTCACCTACTAG	[62]
*P. gingivalis* virulence		
*mfa1*	ATCTTCAGCACTCTCCACAAG TTGTTGGGACTTGCTGCTCTTG	[63]
*kgp*	GCTTGATGCTCCGACTACTC GCACAGCAATCAACTTCCTAAC	[21]
*rgp*	CCGAGCACGAAAACCAA GGGGCATCGCTGACTG	[21]
*luxS*	GAGAGGTGGTTACGACTTTC GTAATCGCCTCGCATCAG	[21]
*ftsH*	CGTCGCAGCATCGCCATCC CAGAGCCTCCGTTGTCGTGATC	[48]
*fimA*	TTGTTGGGACTTGCTGCTCTTG TTCGGCTGATTTGATGGCTTCC	[63]

### Statistical analysis

Data were expressed as mean ± standard deviation (SD) from three independent experiments. Statistical analyses were performed using GraphPad Prism version 6.0 (GraphPad Software, Inc., La Jolla, CA, USA). One-way ANOVA with post hoc Tukey’s test was used in all analyses, and a significance level of 0.05 was established.

## Results

### *Probiotic CFS reduced biomass of mono- and multi-species* P. gingivalis *biofilms*

**Figure 1.**
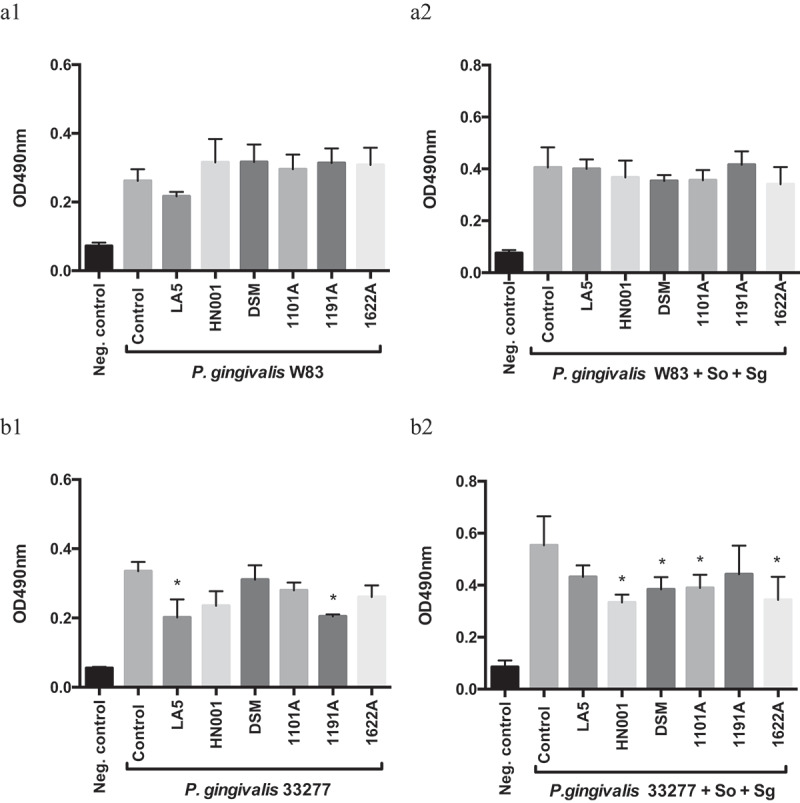
Effect of probiotics cell-free supernatants (CFS) diluted at 1:2.5 on *P. gingivalis* W83 (a1, mono-species; a2, multi-species) and ATCC 33277 (b1, mono-species; b2, multi-species) biofilm biomass, represented by the OD_490nm_ of the standard biofilm dye. Groups: Neg. control- Negative control represents non-inoculated medium; Control- CFS-free positive controls of *P. gingivalis* mono- and multi-species biofilms (So – *S. oralis* and Sg – *S. gordonii*), and experimental group with CFS of: LA5 – *L. acidophilus* LA5, HN001 – *L. rhamnosus* HN001, DSM – *L. reuteri* DSM 17938, 1101A – *B. breve* 110^1A^, 1191A – *B. pseudolongum* 119^1A^ and 1622A – *B. bifidum* 162^2A^. Experiments were conducted in triplicate. (*) Statistically significant difference when compared to respective positive controls using One-way ANOVA with post hoc Tukey’s multiple comparisons (p < 0.05).

Some probiotic CFS reduced mono- and multi-species biofilm biomass of *P. gingivalis* ATCC 33277, but not of strain W83 (Figure 1). However, this reduction was not only dependent on the *P. gingivalis* and probiotic strain, but on the environmental condition (mono- or multi-species biofilm).

### *Probiotic CFS altered* P. gingivalis *proportion in multi-species biofilms*

**Figure 2.**
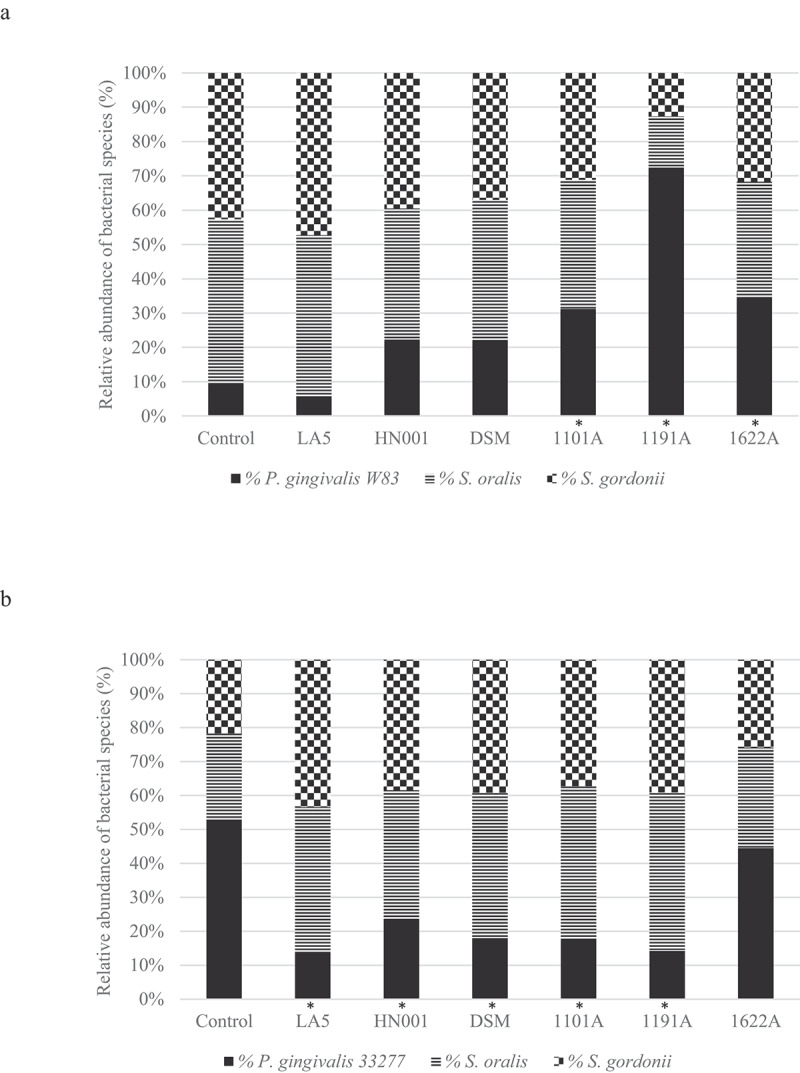
Effect of probiotics cell-free supernatants (CFS of LA5 – *L. acidophilus* LA5, HN001 – *L. rhamnosus* HN001, DSM – *L. reuteri* DSM 17938, 1101A – *B. breve* 110^1A^, 1191A – *B. pseudolongum* 119^1A^ and 1622A – *B. bifidum* 162^2A^) on the relative abundance of *P. gingivalis* W83 (a) or ATCC 33277 (b) and the initial colonizers *S. oralis* and *S. gordonii* in multi-species biofilms, represented as the mean percentage of each bacteria determined by qPCR. (*) Significant difference in *P. gingivalis* counts when compared to respective positive controls using One-way ANOVA with post hoc Tukey’s multiple comparisons (p < 0.05).

**Figure 3.**
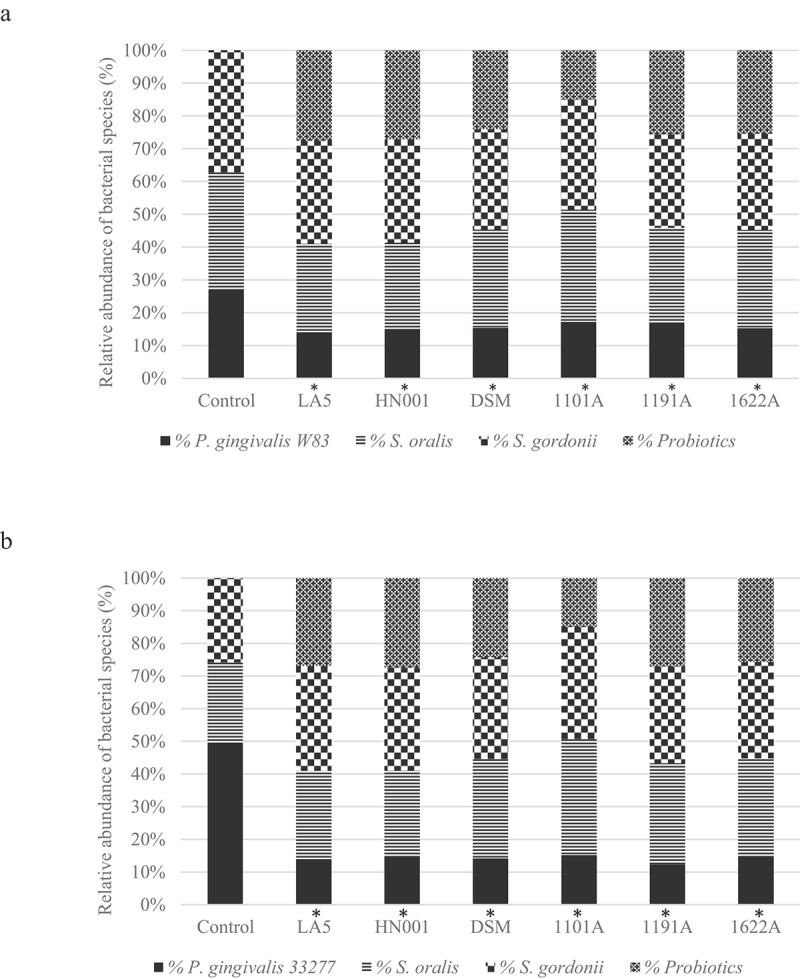
Effect of living *Lactobacillus* sp (*L. acidophilus* LA5, *L. rhamnosus* HN001, *L. reuteri* DSM 17938) and *Bifidobacterium* sp (*B. breve* 110^1A^, *B. pseudolongum* 119^1A^ and *B. bifidum* 162^2A^) on the relative abundance of *P. gingivalis* W83 (a) or ATCC 33277 (b) and the initial colonizers *S. oralis* and *S. gordonii* after cell-to-cell interaction, represented as the mean percentage of each bacteria determined by qPCR (*). Significant difference in *P. gingivalis* abundance, when compared to respective positive controls using One-way ANOVA with post hoc Tukey’s multiple comparisons (p <0.05).

There were no differences in *P. gingivalis* cells number in mono-species biofilms exposed to probiotics CFS (data not shown). Data on absolute number of each bacteria strain/well are shown in supplemental material (Figure S1). Probiotic CFS have altered the relative abundance (%) of *P. gingivalis* in multi-species biofilms in a probiotic/*P. gingivalis* strain-specific manner (Figure 2). CFS of *L. acidophilus* LA5, *L. rhamnosus* HN001, *L. reuteri* DSM, *B. breve* 110^1A^, and *B. pseudolongum* 119^1A,^ but not of *B. bifidum* 162^2A^ decreased the abundance of *P. gingivalis* ATCC 33277. On the other hand, CFS of all tested bifidobacteria, *B. breve* 110^1A^, *B. pseudolongum* 119^1A^ and *B. bifidum* 162^2A^, but not the lactobacilli, increased *P. gingivalis* W83 abundance in multi-species biofilms (Figures 3, and Figure S2 supplemental material, p < 0.05). The relative abundances of *S. oralis* and *S. gordonii* were higher in most groups treated with probiotics CFS in relation to controls in ATCC 33277 multispecies biofilms (p < 00.5) and similar to control in W83 multispecies biofilms (Figure S2 in supplemental material).

### *Living probiotic bacteria reduced* P. gingivalis *abundance in multi-species biofilms*

Probiotic bacteria adhered to biofilm biomass and their abundance in the multi-species biofilms are shown in Figure 3. Data on the number of each strain/well are shown in Figure S3 (supplemental material). Probiotics living cells induced a decrease in *P. gingivalis* abundance (Figure 3, p < 0.05), which was more evident for ATCC 33277 than for W83 (Figure S4, supplemental material). Moreover, most of the living probiotics increased the relative abundance of commensal *S. oralis* and *S. gordonii*) in ATCC 33277 multi-species biofilms and promoted a slight, but significant, decrease in abundance of these commensals in W83 multi-species biofilms (Figures 3 and Figure S4 of supplemental material).

### *Probiotic CFS alter the expression of* P. gingivalis *VAFs in mono- or multi-species biofilms*

Since some probiotic CFS influenced biofilm biomass of *P. gingivalis* ATCC 33277 but not of W83, and altered their abundances in multi-species biofilms, we determined *P. gingivalis* transcription profiles of key VAFs under probiotics CFS. The effect of each CFS on *P. gingivalis* biofilms varied according to the environmental condition, mono- or multi-species biofilm, and VAFs transcription profiles were dependent on the environment. Changes in gene expression were not only related to the studied probiotic species but also dependent on the target *P. gingivalis* strain. Probiotics CFS altered the transcription of genes encoding fimbriae (*mfa1 a fimA*), proteases (*fsH, kgp*, and *rgpA*) and quorum sensing signaling molecules (*luxS*) (Figure 4). Notably, *L. acidophilus* LA5 CFS down-regulated expression of: *mfa1*, in W83 and ATCC 33277 mono-species biofilms (Figure 4a1) and multi-species biofilms (Figure 4a2); *fimA*, in ATCC 33277 (mono-and multi-species); *fsH*, in W83 and ATCC 33277 (mono- and multi-species); *kgp*, in W83 (mono-species) and ATCC 33277 (mono- and multi-species); *rgp*, in W83 and ATCC 33277 (mono-species); and *luxS*, in W83 (mono-species) and ATCC 33277 (mono- and multi-species) (Figure 4).

**Figure 4.**
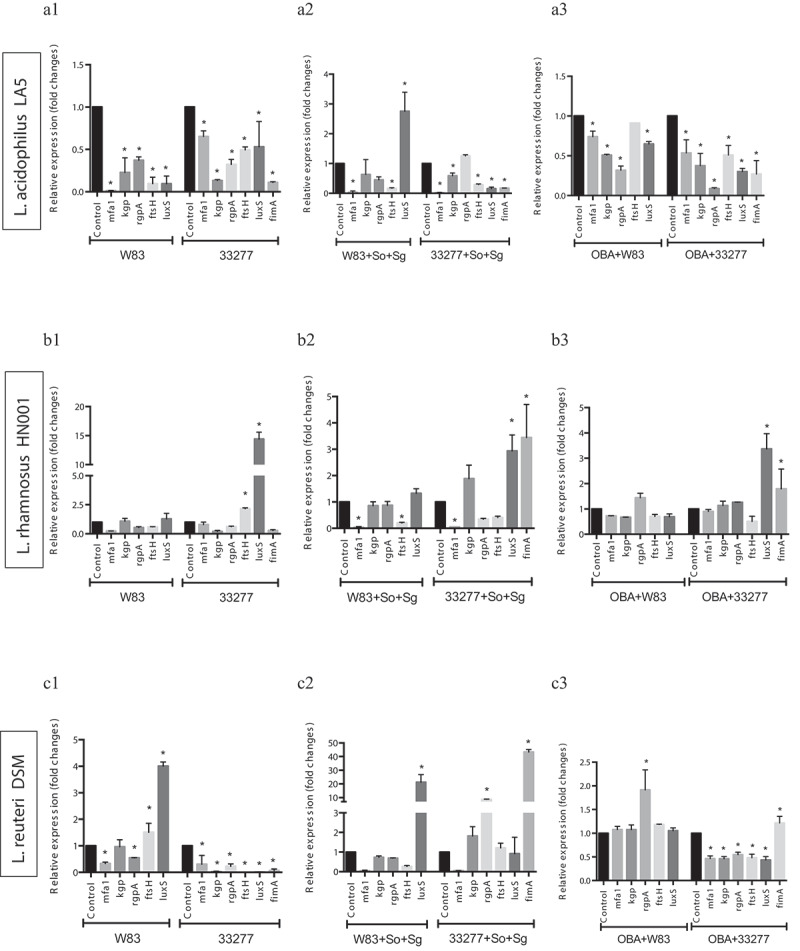
Effect of probiotics on the relative transcription of *P. gingivalis* encoding virulence genes (mfa1 – minor fimbriae; fimA – major fimbriae; kgp – lysine gingipain; rgpA – arginine gingipain; ftsH – metalloproteinase, and luxS – quorum sensing components), determined by RT-qPCR. *P. gingivalis* strains W83 and ATCC 33277 biofilms formed in BHIHM broth added with the supernatant of cultures of probiotics (a1 and a2 - *L. acidophilus* LA5, b1 and b2 – *L. rhamnosus* HN001, c1 and c2 – *L. reuteri* DSM 17938, d1 and d2 – *B. breve* 110^1A^, e1 and e2 – *B. pseudolongum* 119^1A^ and f1 and f2 – *B. bifidum* 162^2A^) diluted to 1:2.5, in mono-species (a1, b1, c1, d1, 1 and f1) and multi-species (a2, b2, c2, d2, e2 and f2), and infecting OBA-9 GECs with probiotic co-infection at a MOI of 1:1,000 (a3 - *L. acidophilus* LA5, b3 – *L. rhamnosus* HN001, c3 – *L. reuteri* DSM 17938, d3 – *B. breve* 110^1A^, e3 – *B. pseudolongum* 119^1A^ and f3 – *B. bifidum* 162^2A^). Data are expressed as fold changes in relation to positive control conditions (biofilms without probiotic cell-free supernatants or GECs without probiotic bacteria), after normalization to the endogenous control gene *16SrRNA*. (*) Significant difference when compared to respective positive controls using One-way ANOVA with post hoc Tukey’s multiple comparisons (p < 0.05).

**Figure 4.**
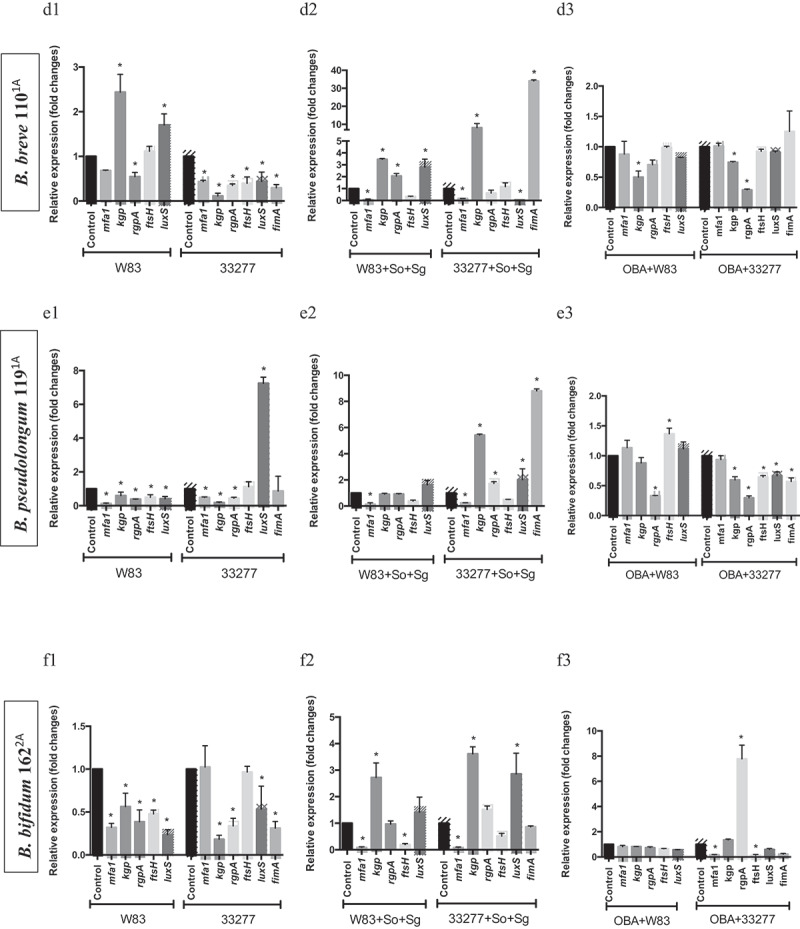
(Continued).

### *Living probiotics alter the expression of* P. gingivalis *VAFs under gingival epithelial cell co-infection*

Our previous study [9] reported that living *Lactobacillus* and *Bifidobacterium* reduced *P. gingivalis* ATCC 33277 and W83 adhesion and invasion of GECs, except for *Lactobacillus* which did not reduce invasion by strain W83. Moreover, these probiotics decreased pro-inflammatory cytokines synthesis induced by *P. gingivalis*. Data on the expression analysis of *P. gingivalis* VAF genes in co-infection with probiotic bacteria in GECs revealed that most studied genes were regulated by at least one probiotic, and the transcription profiles were probiotic-strain and *P. gingivalis*-strain specific (Figure 4a3,b3,c3,d3,e3,f3). *L acidophilus* LA5 showed prominent activity on VAF transcription, including the downregulation of the fimbriae encoding genes *mfa1*, in both strains of *P. gingivalis*, and *fimA*, in ATCC 33277 (Figure 4a3); gingipains encoding genes *kgp, rgpA*, and the quorum sensing *luxS* in ATCC 33277 or W83 interacting with GECs (Figure 4).

## Discussion

An ideal probiotic to control periodontitis should keep the balance between the oral biofilm and the host by controlling pathogens colonization and modulating the inflammatory response. On this basis, we have recently shown that the studied probiotics were able to reduce *P. gingivalis* ATCC 33277 and W83 adherence to and invasion of GECs as well as to modulate the epithelial cell immune response against this periodontopathogen [9]. Altogether, our present and previous data indicated that soluble by-products and living probiotic strains reduced *P. gingivalis* abundance in multi-species biofilms as well as its interaction with GECs. We have also shown that the probiotics can alter the transcription profile of *P. gingivalis* virulence-associated factors, thus interfering in its ability to colonize the host and subvert immune response.

Probiotics CFS showed little or no effect on the biomass of *P. gingivalis* mono-species biofilms. However, when tested on multi-species biofilms, which simulates a more realistic scenario in the oral cavity, some probiotics living cells and/or their soluble released products in the culture medium (CFS) reduced the abundance of *P. gingivalis* without little effect on the relative abundance of early colonizers, such as *S. oralis* and *S. gordonii*. Dysbiosis rate is referred to as the ratio between the relative abundances of disease-associated species to that of health-associated species [29]. Hence, the selective effect of probiotics on the pathogen suggests that probiotics would reduce the dysbiosis in the oral biofilms. Furthermore, our results raise the hypothesis that *P. gingivalis* co-aggregation mechanisms should be disturbed by certain probiotics, since streptococci comprise 70% of the initial colonizers that interact with proteins/receptors on the acquired pellicle [30], and the ability of *P. gingivalis* to form biofilms is dependent on co-aggregation with such bacteria [31].

Nevertheless, *P. gingivalis* presents a high diversity as indicated by their different virulent surface components [32]. *P. gingivalis* W83 is a capsulated/afimbriated highly virulent strain, whereas ATCC 33277 is a no capsulated/fimbriated less virulent strain [32,33]. These *P. gingivalis* strains also differ in relation to biofilm formation [34] and chromosomal transposable elements, which may alter their gene transcription [35], and physiology, such as their capacity to survive under oxidative stress [36], which altogether can contribute to explain differences on biofilm composition and transcription of virulence factors induced by probiotics.

Interestingly, gene regulation of the two *P. gingivalis* strains promoted by the probiotics differed under different environmental conditions such as in mono-species and in multi-species biofilms. Recent data reported that *P. gingivalis* W83 causes a more severe dysbiosis than *P. gingivalis* ATCC 33277 in a multispecies biofilm model [23]. On the other hand, transcriptome analysis reported that each strain differed in their ability to modulate the expression *S. mitis* genes [23]. Our data contribute to this discussion, showing that products released by the commensal *B. pseudolongum* 119^1A^ may reduce the dysbiosis promoted by strain ATCC 33277 but not by W83. Furthermore, not only *P. gingivalis* alters the transcription profile of commensals as shown previously [23] but also the ability of commensal lactobacilli and bifidobacteria to alter the transcription profile of *P. gingivalis* was also dependent on the pathogen strain.

Our data have also indicated that lactobacilli and bifidobacteria may participate in *in vitro* multi-species biofilms with *P. gingivalis* and streptococci, and most inhibitory effects were obtained with living cells-to-cell interaction. Previous study, also adopting a multi-species biofilms model, reported that bifidobacteria induced a reduction in *P. gingivalis* levels [37]. *Lactobacillus* spp. and *Bifidobacterium* spp. are part of the normal resident microbiota, and some strains of these genera such as *L. acidophilus, L. rhamnosus* and *B. longum* produce exopolysaccharides (EPS) that contribute to biofilm formation [38]. Thus, our data suggest that interactions between probiotics surface components such as extracellular polysaccharides and surface-layer proteins with commensal bacteria [39,40] may compete with *P. gingivalis* W83 adhesion/aggregation mechanisms.

Virulence of *P. gingivalis* is mediated by an array of factors involved in several functions, such as attachment to host surfaces and other oral microorganisms, acquisition of nutrients, induction of a destructive inflammatory response and evasion of host response. Probiotics were not only able to decrease the abundance of *P. gingivalis* in multispecies biofilms but were also able to inhibit adhesion and invasion of *P. gingivalis* to GECs [9]. Once again, these activities were dependent on the *P. gingivalis* strain, since the tested lactobacilli were able to inhibit *P. gingivalis* W83 adhesion to but not the invasion of GECs, whereas both adhesion and invasion of ATCC 33277 were inhibited.

In order to shed some light on the inhibitory mechanisms of probiotics, we have shown that these beneficial bacteria were able to alter the transcription of genes associated with several virulence-associated factors. Lactobacilli and bifidobacteria overall down-regulated the expression of *fimA*, which encodes the main fimbriae FIMA in *P. gingivalis* ATCC 33277. These fimbriae provide pathogen binding to saliva and serum components, and to extra-cellular matrix proteins and epithelial cells [41,42]. It also promotes auto-aggregation and co-aggregation to other bacterial species, including *S. gordonii* [43] and reasonably its down-regulation can explain the reduction in *P. gingivalis* abundance in multi-species biofilm. Moreover, FIMA is associated with pathogen adhesion/invasion to epithelial cells [44] and it is recognition by host cells through TLR2 and TLR4 receptors with downstream activation of pro-inflammatory cytokines [45]. Thus, *fimA* down-regulation may explain our previous study that found a modulation of the immune response triggered in gingival epithelial cells followed by a reduction in the adhesion of *P. gingivalis* ATCC 33277 [9]. Curiously, all tested probiotics supernatants were able to down-regulate the expression of *mfa1* in multi-species biofilm assays. The minor fimbriae MFA1 is also involved in *P. gingivalis* auto-aggregation [46] and co-aggregation with *S. gordonii* [47]. Thus, a reduction in *mfa1* transcription promoted by probiotic secretome would impair *P. gingivalis* interaction with streptococci.

Probiotics and their by-products were also able to regulate transcription of proteases encoding genes. FstH is an integral membrane zinc metallopeptidase that participates in a network that restrains biofilm accumulation in heterotypic *P. gingivalis* ATCC 33277-*S. gordonii* biofilms [48]. Thus, alteration in this regulatory mechanism promoted by probiotics would alter *P. gingivalis* interaction with *S. gordonii* in multi-species biofilms.

Our data have also revealed that some probiotics down-regulated the transcription of *kgp* and *rgp* that encode lysine- and arginine-gingipains, respectively, in W83 and ATCC 33277 strains. Lysine-gingipain (Kgp) degrades tissue matrix and proteins that contain iron and hemin [21,49], and arginine-gingipain (Rgp, cysteine protease) regulates exopolysaccharide accumulation, and promotes hemagglutination and maturation of several *P. gingivalis* surface proteins such as fimbrilin of FIMA [14,20]. Negative regulation of *kgp* and *rgpA* transcription in W83 was achieved in most conditions by *L. acidophilus* LA5, whereas other probiotics even up-regulated transcription of these proteases encoding-genes. In fact, a proteomics of *L. acidophilus* revealed that an increased cysteine synthase activity may accumulate a cysteine pool relevant for protein stability and enzyme catalysis in this probiotic, which suggests its advantage in terms of protease regulation [50]. Still, the decreased expression of *kgp* and *rgp* induced by some probiotics, especially *L. acidophilus* LA5, may not only impair uptake of iron and decrease tissue destruction by the periodontopathogen strains but should also play an important role adherence of *P. gingivalis* to epithelial cells [20,51], in consonance with our previous data [9]. Furthermore, several surface components of bacteria are proteases-sensitive, including those of probiotics [39,40], and they could be degraded by *P. gingivalis* proteolytic activity [52]. Hence, regulation of gingipains encoding genes *kgp* and *rgpA* may have influenced the effect of probiotics on biofilm formation by different *P. gingivalis* strains.

On the other hand, the effects of probiotics on the expression of *luxS* were contradictory. Such findings indicate differences in the regulatory mechanisms of transcription between *P. gingivalis* W83 and ATCC 33277. LuxS controls quorum sensing, a system in response to bacterial biofilm density through the release of auto-inducers (AI-2) [21]. Different microorganisms use this system to communicate with each other, and AI-2 works like a ‘universal language’ for intra-species and inter-species communication. LuxS/AI-2 of *P. gingivalis* regulates proteinase and hemagglutinin activities [21,53,54] and may affect the expression of biofilm-associated genes, such as fimbriae [14]. Thus, a beneficial effect of *L. acidophilus* LA-5 could be associated with the down-regulation of transcription of *P. gingivalis luxS* in mono-species biofilms. However, LuxS is also expressed by probiotics, influencing their ability to adhere, produce exopolysaccharides and form biofilm [55,56]. In addition, LuxS/AI-2 from *Bifidobacterium* spp. plays an essential role in their metabolism by regulating iron acquisition [57] but data on probiotic lactobacilli are sparse [58]. Interestingly, quorum-sensing signaling promoted by commensal microorganisms may disturb pathogen communication, since *P. gingivalis* LuxS/AI-2 network is reduced by the AI-2 synthetized by *S. gordonii* [59], which consequently points to a beneficial effect of increasing LuxS expression in multi-species biofilms as observed under *L. acidophilus* LA-5 secretome stimulus. Therefore, since *luxS* involves a system common to pathogens, probiotics and other commensals, distinct results would be expected, depending on the environmental conditions and evaluated strains.

In summary, probiotics may affect biofilm formation and adherence of *P. gingivalis* to host cells by regulating the transcription of its virulence-associated factors. However, their effects differ not only regarding the probiotic species but also according to the colonizing pathogen strain. Taken all together, *L. acidophilus* LA 5 exerted the most promising effects by its secretome or by direct probiotic-pathogen contact that reduced *P. gingivalis* proportion in multi-species biofilms and promoted overall down-regulation transcription of key virulence genes responsible for encoding fimbriae, proteases, and quorum sensing components. Although bacterial interaction plays a key role in establishing the oral microbial communities, most of the interaction between *P. gingivalis* and probiotics are poorly understood. None of the studied probiotic strains was well characterized and further evaluation on the probiotics composition and released products should be performed, in order to elucidate their distinct mechanisms on the interaction with *P. gingivalis*.

Further *in vivo* experimental models and clinical studies are needed to provide evidence on the potential of these probiotics, especially *L. acidophilus* LA5, to control periodontopathogens levels and tissue destruction in the context of the periodontitis.

## Supplementary Material

Supplemental Material
