# Supplementary material for: Probiotics alter biofilm formation and the transcription of Porphyromonas gingivalis virulence-associated genes
Source: J Oral Microbiol. 2020 Aug 20;12(1):1805553. doi: 10.1080/20002297.2020.1805553 (PMC7482675; doi:10.1080/20002297.2020.1805553)
Supplement: Supplemental Material [file ZJOM_A_1805553_SM3419.zip › Supplementary/Supplemental material .docx]

Supplemental material

1. **Effect of probiotics CFS on multi-species biofilm.**

Figure 1s. Effect of probiotics cell-free supernatants (CFS) diluted at 1:2.5 on multi-species biofilms formed by *P. gingivalis* W83 or ATCC 33277, *S. oralis* and *S. gordonii*. Amount of each bacterial specie is represented by the absolute number of cells /well (mean ± standard deviation). Control- CFS-free positive controls of *P. gingivalis* W83 or ATCC 33277 multi-species biofilms (So – *S. oralis* and Sg – *S. gordonii*), and experimental group with CFS of: LA5 – *L. acidophilus* LA5, HN001 – *L. rhamnosus* HN001, DSM – *L. reuteri* DSM 17938, 1101 – *B. breve* 110^1A^, 1191 – *B. pseudolongum* 119^1A^ and 1622 – *B. bifidum* 162^2A^. Experiments were conducted in triplicate. (*) Significant difference in bacteria counts when compared to respective positive controls, using One-way ANOVA with post hoc Tukey's multiple comparisons (p <0.05).

Figure 2s. Effect of probiotics cell-free supernatants (CFS) diluted at 1:2.5 on multi-species biofilms formed by *P. gingivalis* W83 or ATCC 33277, *S. oralis* and *S. gordonii*. Amount of each bacterial specie is represented by the relative abundance in percentage (mean ± standard deviation). Control- CFS-free positive controls of *P. gingivalis* W83 or ATCC 33277 multi-species biofilms (So – *S. oralis* and Sg – *S. gordonii*), and experimental group with CFS of: LA5 – *L. acidophilus* LA5, HN001 – *L. rhamnosus* HN001, DSM – *L. reuteri* DSM 17938, 1101 – *B. breve* 110^1A^, 1191 – *B. pseudolongum* 119^1A^ and 1622 – *B. bifidum* 162^2A^. Experiments were conducted in triplicate. (*) Significant difference in bacteria counts when compared to respective positive controls, using One-way ANOVA with post hoc Tukey's multiple comparisons (p <0.05).

1. **Effect of living probiotics on multi-species biofilm.**

Figure 3s. Effect of probiotics living cells on multi-species biofilms formed by *P. gingivalis* W83 or ATCC 33277, *S. oralis* and *S. gordonii*. Amount of each bacterial specie is represented by the absolute number of cells /well (mean ± standard deviation). Control- *P. gingivalis* W83 or ATCC 33277 multi-species biofilms (So – *S. oralis* and Sg – *S. gordonii*), and experimental group with living cells of: LA5 – *L. acidophilus* LA5, HN001 – *L. rhamnosus* HN001, DSM – *L. reuteri* DSM 17938, 1101 – *B. breve* 110^1A^, 1191 – *B. pseudolongum* 119^1A^ and 1622 – *B. bifidum* 162^2A^. Experiments were conducted in triplicate. (*) Significant difference in bacteria counts when compared to respective positive controls, using One-way ANOVA with post hoc Tukey's multiple comparisons (p <0.05).

Figure 4s. Effect of probiotics living cells on multi-species biofilms formed by *P. gingivalis* W83 or ATCC 33277, *S.oralis* and *S. gordonii*. Amount of each bacterial specie is represented by the relative abundance in percentage (mean ± standard deviation). Control- *P. gingivalis* W83 or ATCC 33277 multi-species biofilms (So – *S. oralis* and Sg – *S. gordonii*), and experimental group with living cells of LA5 – *L. acidophilus* LA5, HN001 – *L. rhamnosus* HN001, DSM – *L. reuteri* DSM 17938, 1101 – *B. breve* 110^1A^, 1191 – *B. pseudolongum* 119^1A^ and 1622 – *B. bifidum* 162^2A^. Experiments were conducted in triplicate. (*) Significant difference in bacteria counts when compared to respective positive controls, using One-way ANOVA with post hoc Tukey's multiple comparisons (p <0.05).
