# Supplementary figures and images for: Probiotics alter biofilm formation and the transcription of Porphyromonas gingivalis virulence-associated genes
Source: J Oral Microbiol. 2020 Aug 20;12(1):1805553. doi: 10.1080/20002297.2020.1805553 (PMC7482675; doi:10.1080/20002297.2020.1805553)

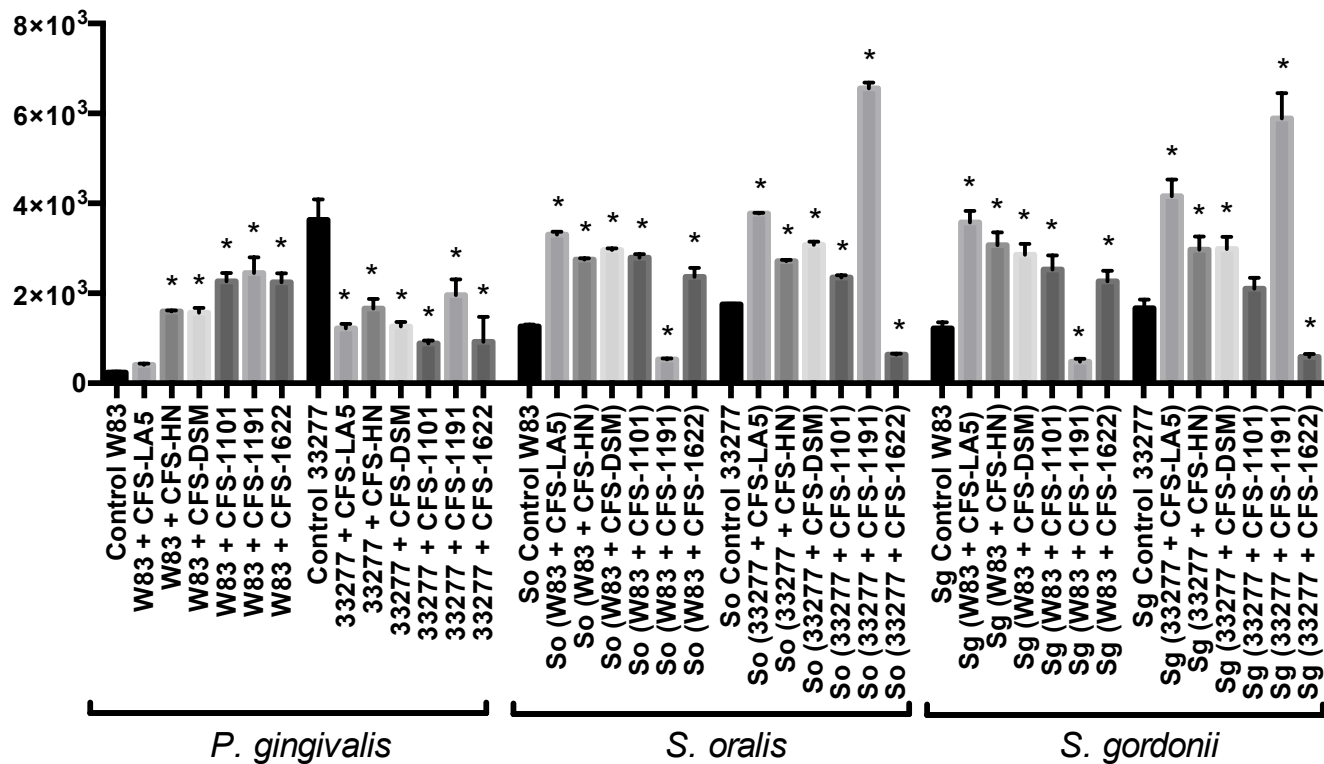

Supplement: Supplemental Material [file ZJOM_A_1805553_SM3419.zip › Supplementary/Figure 1s..pdf]

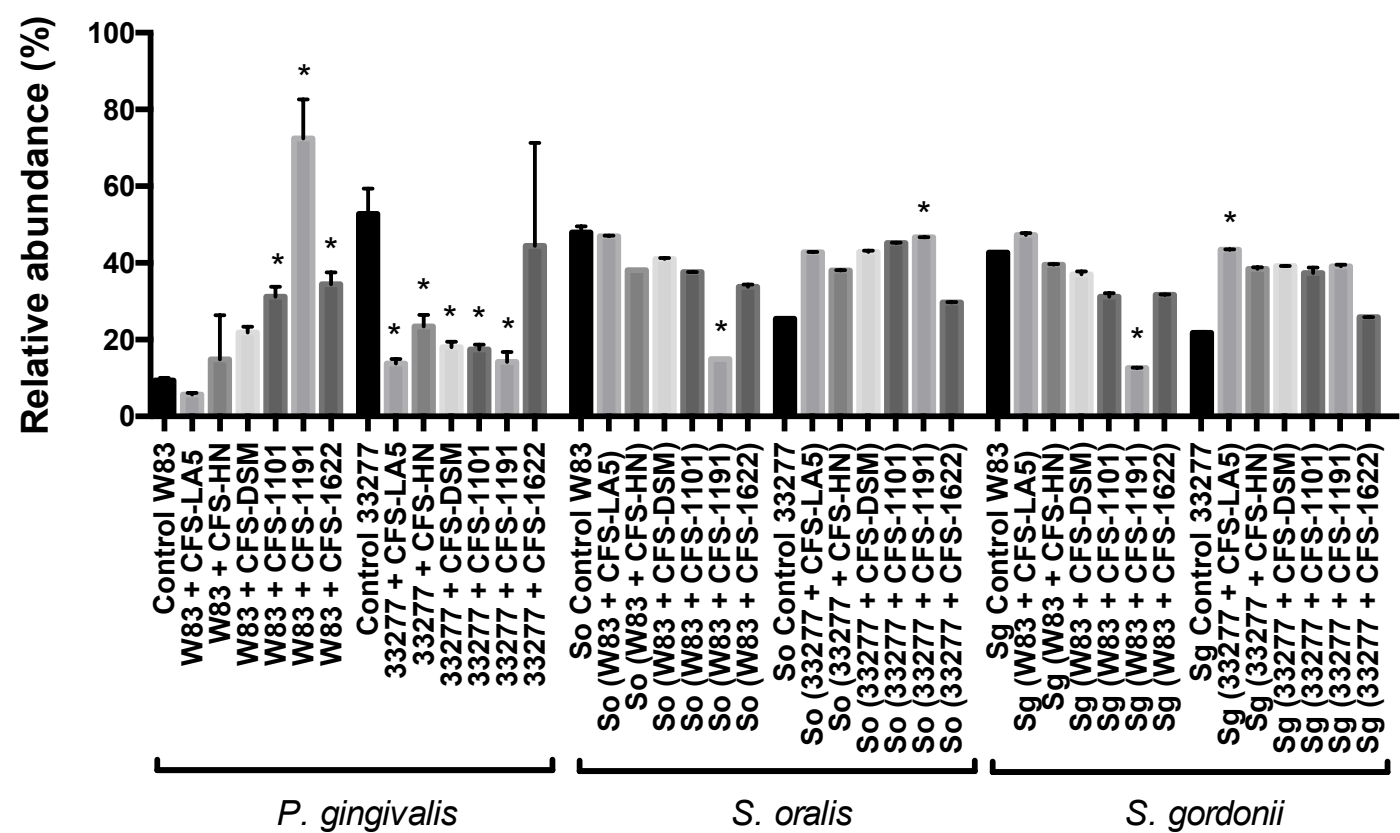

Supplement: Supplemental Material [file ZJOM_A_1805553_SM3419.zip › Supplementary/Figure 2s. .pdf]

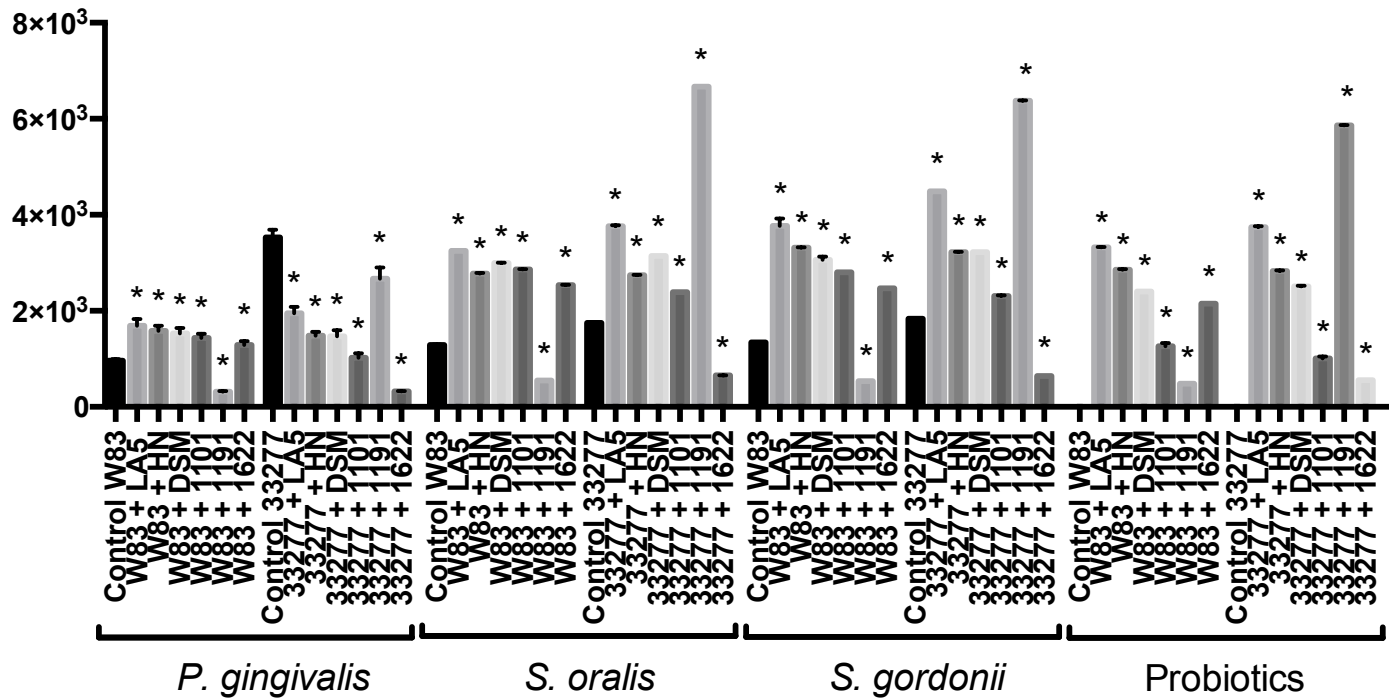

Supplement: Supplemental Material [file ZJOM_A_1805553_SM3419.zip › Supplementary/Figure 3s..pdf]

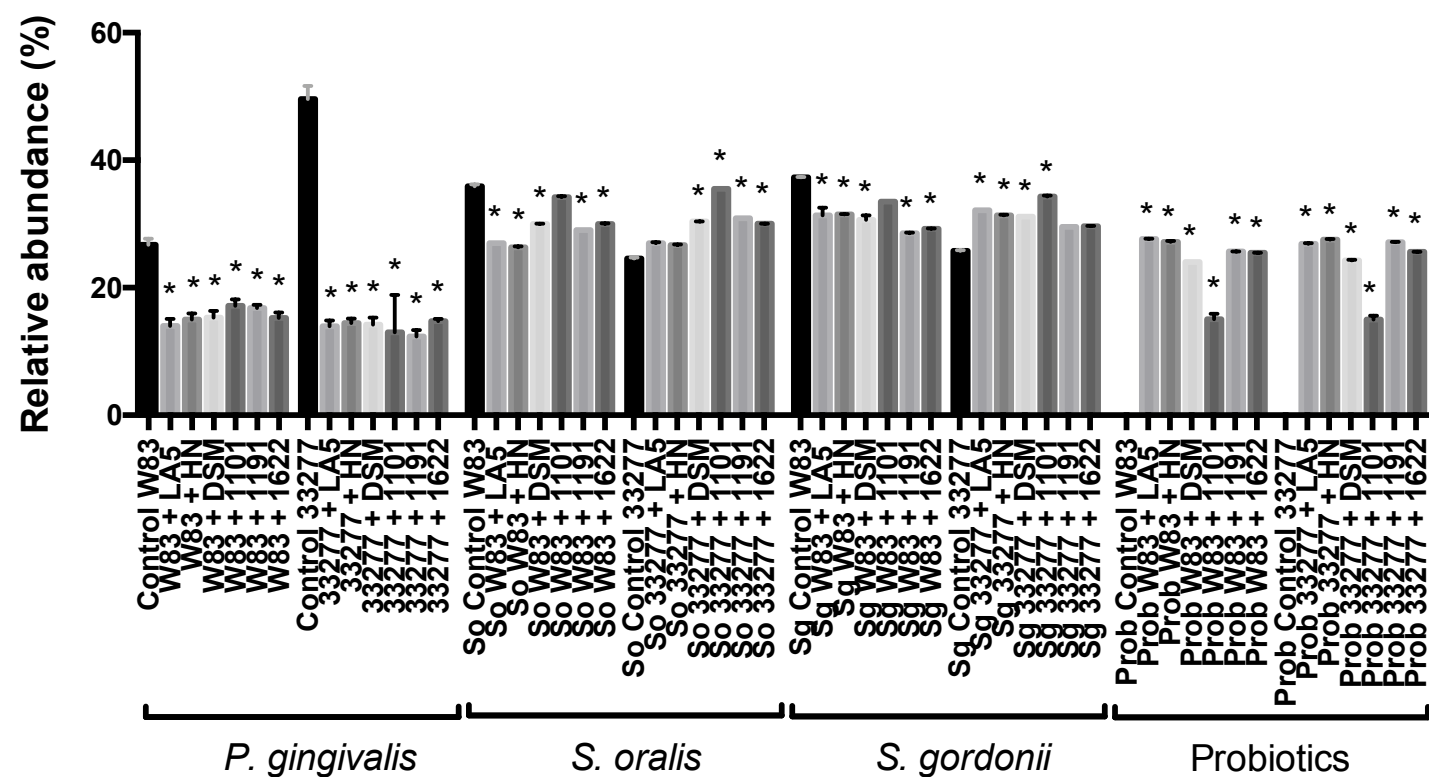

Supplement: Supplemental Material [file ZJOM_A_1805553_SM3419.zip › Supplementary/Figure 4s..pdf]
